# Supplementary material for: Molecular epidemiology of Mycobacterium tuberculosis complex in the Volta Region of Ghana
Source: PLoS One. 2021 Mar 17;16(3):e0238898. doi: 10.1371/journal.pone.0238898 (PMC7968653; doi:10.1371/journal.pone.0238898)
Supplement: Attachment — Submitted filename: Response to reviewers.doc [file pone.0238898.s001.doc]

Dear Editor,

Please find attached a revised version of our joint manuscript “**Molecular epidemiology of *Mycobacterium tuberculosis* complex in the Volta Region of Ghana**”, which we would like to resubmit for publication in your esteemed journal PLOS ONE.

Data cannot be shared publicly because of patient confidentiality. Data are available from the Noguchi Memorial Institutional Data Access / Ethics Committee (contact via [GWemakor@noguchi.ug.edu.gh](mailto:GWemakor@noguchi.ug.edu.gh)) for researchers who meet the criteria for access to confidential data.

We appreciate the time and efforts by the editor and referees in reviewing this manuscript. We have addressed all issues indicated in the review report in a Point by Point table below.

Thank You

Yours sincerely,

Adwoa Asante-Poku, PhD

Tel. (+233)302940421/22; Fax (+233) 302502182

Address: Noguchi Memorial Institute for Medical Research, Bacteriology Department. P. O. Box LG 581, Legon, Accra-Ghana.

Email : aasante-poku@noguchi.ug.edu.gh

Editors Comments

| **Comment** | **Response** | **Line** |
| --- | --- | --- |
| 1.Please ensure that that your manuscript meets PLOS ONE's style requirements, including those for file naming. | The Manuscript has been revised to meet PLOS ONE style requirement |  |
| 2. Thank you for your title change from "phylogenetic..." to "molecular epidemiology..." Please also revise the text of your manuscript to remove references to phylogenetics in the following places: a) KeyMessage b) Last paragraph of your Introduction c) End of your Genotyping of MTBC section d) Figure 2 legend | The Manuscript text has been revised to remove references to “phylogenetic in the key areas suggested |  |
| 3.We note that you have indicated that data from this study are available upon request. PLOS only allows data to be available upon request if there are legal or ethical restrictions on sharing data publicly. For information on unacceptable data access restrictions, please see [http://journals.plos.org/plosone/s/data-availability#loc-unacceptable-data-access-restrictions](http://journals.plos.org/plosone/s/data-availability#loc-unacceptable-data-access-restrictions).  In your revised cover letter, please address the following prompts:  a) If there are ethical or legal restrictions on sharing a de-identified data set, please explain them in detail (e.g., data contain potentially identifying or sensitive patient information) and who has imposed them (e.g., an ethics committee). Please also provide contact information for a data access committee, ethics committee, or other institutional body to which data requests may be sent.  b) If there are no restrictions, please upload the minimal anonymized data set necessary to replicate your study findings as either Supporting Information files or to a stable, public repository and provide us with the relevant URLs, DOIs, or accession numbers. Please see <http://www.bmj.com/content/340/bmj.c181.long> for guidelines on how to de-identify and prepare clinical data for publication. For a list of acceptable repositories, please see [http://journals.plos.org/plosone/s/data-availability#loc-recommended-repositories](http://journals.plos.org/plosone/s/data-availability#loc-recommended-repositories).  We will update your Data Availability statement on your behalf to reflect the information you provide. | The cover letter has been revised to reflect the ethical restrictions on the data set. |  |
| Please include a copy of Table 5 which you refer to in your text on page 12. | Table 5 has been removed from the manuscript accordingly |  |

**Reviewers Comments**

**Reviewer #1**

| **Reviewers comment** | **Response** | **Line** |
| --- | --- | --- |
| Ensure that formatting and grammar is checked throughout the entire document, there are several grammatical errors throughout the document, i.e. lines 37 and 44, no full stops after a sentence, line 73 two commas after Ethiopia, Line 78 check spacing before and after 7 | We thank the reviewer for the discovery of these errors.  The grammatical errors throughout the document corrected accordingly |  |
| Abstract: Instead of the statement "standard methods" list the methods used during analysis refer to line 42.  Decimal places should be consistent throughout the manuscript i.e. line 49 is presented as 76.04% instead of 76.06% which should be rounded to 76.1% to maintain one decimal place throughout the manuscript.  Check that the calculations for percentages are correct, percentages are not adding up to 100%, refer to lines 49 and 50.  List percentages for the two orphans.  It will be useful to add Ghana and spoligotyping to the list of key words and either limit to tuberculosis and MAF in order to maintain the journal limit. | Standard methods haven clarified.  Decimal places corrected throughout the manuscript to one decimal place  All percentages corrected throughout the manuscript to one decimal place  Percentages of the two orphans listed  Ghana and spoligotyping added to the key words | Line 41-42  Line 49  Line 52  Line 52  Line 58 |
| Some statements will need to be rephrased in order for them to be more cohesive i.e.  lines 68/69 rephrase the sentence beginning "The worldwide." to "Due to the worldwide".  Insert "TB"after multidrug-resistant in line 68.  Rephrase line 71 to "...public health challenge; with a TB incidence rate of...".  Check line 72 "a is ranked...".  Line 74 insert "and" between Uganda and South Africa, delete "the" before Africa in line 74 | The worldwide corrected Due to the worldwide"  "TB" inserted after multidrug-resistant  Line 70 corrected to read: "with a TB incidence rate of".  "a" deleted  and" inserted between Uganda and South Africa | Line 70  Line 70  Line 73  Line 74  Line 76 |
| Study design: line 136 should be changed from "on patients" to "of patients". Line 137,  state how many samples were included, were all samples included? and how samples were selected for analysis.  line 150, there shouldn't be a space between 37 and degree C.  DRb and not "Drb"line 165.  Spacing between bovis and BCG line 167.  lines 171 to 174 "in order... each blot" are not necessary, authors can delete the statement and simply refer to the reference.  Line 175 should make reference to the database use to assign SIT and orphans. line 178 refers to the SpolDB4, however the SITVIT2 is a more updated version of the SpolDB4 which the authors should use to compare their spoligo findings and make reference to. Authors should cite manufacturers correctly line 188. | line 136 changed from "on patients" to "of patients"  All samples collected were included in the study  Space between 37 and degree C corrected  "Drb" corrected to DrB  Spacing between bovis and BCG corrected  Text in Lines 171 to 174 deleted  Obtained spoligotyping patterns were compared with those available in the international spoligotype database SITVIT and SpolDB4 | Line 142  Line 142  Line 155  Line 174  Line 176  Line 212 |
| Data analysis: the titles data analysis and results (lines 192 and 201) are underlined which is not consistent with the remainder of the manuscript.  Was SITVIT2 or SpolDB not used for analysis? line 197. | We have duly undone the underlines  Both SITVIT2 and SpolDB were used for analysis | Line 207  Line 233  Line 212 |
| Results: The sample size should also be mentioned earlier, somewhere in the study design i.e. line 137.  Line 204, only 125 out of 270 (46%) positive isolates were characterised, why is this the case? This impacts on the conclusions drawn as it is not a good representation of isolates from this region, should be mentioned later as a limitation to the study.  Delete "The" from the title in line 216.  Was phenotypic DST data available? If so this should be included in table 1.  Lines 218-219, lineage statistics don't add up to 100% and are not matching with the data presented in the abstract.  Line 244 table number not indicated.  Line 252 "wide type band" shouldn't be in italics. | sample size indicated in the study design  Thank you very much for the comment. However, 270 participants were enrolled into the study. One hundred and eighty-three were confirmed as acid-fast bacilli positive (67.8%), of which 125 culture positives (68%) where obtained. The results has been corrected to reflect this statement.  "The deleted from the Title  DST was performed for all isolates obtained. The results has been presented in a Table 3  Percentages corrected to match the data presented in the abstract  Table number indicated  "wild-type band" has been un- italised | Line 142  Line 235-239  Line 249  Line 300  Line 258  Line 258  289 |
| Discussion: line 278, refrain from listing i), ii)...  Line 287 should read "Approximately..." and not "The approximately...".  A full stop after ref (14) in line 287.  Line 289, it is not advisable to start a sentence with an abbreviation "MAF".  In your discussion and conclusion you will need to address the fact that only 46% of isolates collected over the study period were characterised and how this would impact on the data that you have presented.  Line 310, delete "pulmonary" since drug resistant TB can impact treatment outcome EPTB as well as PTB.  et al should be in italics throughout including in reference list.  Abbreviate M. abscessus in line 333 unless this was the first use.  Rephrase the statement "The challenge is, microscopy..."in line 335.  There is a big jump from speaking about diagnostics to concluding on MAF genotype, you will need to add a few sentences that connect the previous statement with your conclusion line 341. | Numbering of key results has been deleted  Line 291 corrected accordingly  Full stop added accordingly  MAF written out in full  Thank you very much for the comment. However, 270 participants were enrolled into the study. One hundred and eighty-three were confirmed as acid-fast bacilli positive (67.8%), of which 125 culture positives (68%) where obtained. The results has been corrected to reflect this statement  As suggested by reviewer "pulmonary" deleted from the sentence  *et al* italised throughout the reference list  *Mycobacterium abscessus* abbreviated to M. *abscessus* in text  The sentence has been rephrased | Line 311-314  Line 325  Line 320  Line 293  Line 248 -250  Line 410  Line 377  Line 435 |
| References: Check the reference style is uniform throughout your reference list and conforms with the journal requirements, italicize "et al". Delete () in line 373 | Reference style corrected  () in Line 450 | Line 515 |
| Figures: Is figure 1 the authors own work? If not cite the source. Figure 2 percentages are not adding up to 100%. | Thank you for the comment. Figure 1 is the authors own work |  |

**Reviewer #2**

| **Reviewers comment** | **Response** | **Line** |
| --- | --- | --- |
| you need to adjust the space between some words see line 37;44;73;78;99;167:204;207;228;235;244;273;313;319 | Topographic errors have been corrected through out the manuscript |  |
| Please make *M.africanum* in italic in all the manuscript for example line 62. | *M.africanum* has been italised through the manuscript | Line 62 |
| Also a coma must be place in line 260 after table 3 and in line 287 after the refererence 14 | Thank you for the comment. A full stop has been placed in Line 266 after Table 3 and Line 287 | Line 295, Line 320 |
| some references are old like the TB report which date is 2018, new TB report are available please use it | New TB report used |  |
| References content some errors which need to be correct: references 2;4;6;9;10;12;16;25;29;30;31 | Reference content corrected |  |
| Table 3 must be redone to improve understanding because it is rather poorly presented | Thank you for the comment. Table 3 has been redone | Line 300 |
